# Promoting health protection worldwide: The International Labour Organisation and health systems financing, 1952–2012

**DOI:** 10.1080/07075332.2019.1582550

**Published:** 2019-04-02

**Authors:** Christopher Sirrs

**Affiliations:** Centre for History in Public Health, London School of Hygiene and Tropical Medicine, London, UK

**Keywords:** International Labour Organisation, health systems, universal health coverage, social health insurance, international development

## Abstract

In recent years, universal health coverage (UHC) has returned with a vengeance to the international agenda, raising complex and highly political questions about how health systems should be organised and financed. Drawing upon an extensive analysis of archival material, this article examines the International Labour Organisation’s (ILO) approach towards health systems financing in the second half of the twentieth century, exploring its evolving strategy towards social health protection in the context of international development, and its relationship with other international agencies, notably the World Health Organisation and World Bank. It argues that while the ILO’s role in international development has come into question in recent decades, its officials have nevertheless made a meaningful contribution to the promotion of health protection worldwide. Despite the wider marginalisation of universalism in post-war international discourse, ILO officials continually shifted their strategy to ensure that mechanisms of health protection such as social health insurance were prioritised in health systems development. ILO support contributed to some notable successes, such as the achievement of UHC in Thailand in 2002.

## 1. Introduction

In September 2012, Judith Rodin, President of the Rockefeller Foundation, and David de Ferranti, President of the Results for Development Institute, opened a new series of *The Lancet* by declaring how ‘a third great transition seems to be sweeping the globe, changing how health care is financed and how health systems are organised.’^1^ Alluding to the demographic and epidemiological transitions of the 18th–20th centuries, the transition Rodin and de Ferranti were referring to was the dramatic resurgence of international interest in universal health coverage (UHC): population-wide access to health services and financial risk protection against the costs of illness. Since 2005, the World Health Organisation (WHO) has laid out a path to UHC, recognising that, while there is no one-size-fits-all solution, basic health services and financial risk-protection mechanisms are vital to improve health equity.^2^ A United Nations (UN) General Assembly Resolution in 2012 called on governments ‘to urgently and significantly scale up efforts to accelerate the transition towards universal access to affordable and quality health-care services’.^3^ An International Labour Organisation (ILO) recommendation that same year urged governments to strengthen national floors of social protection, including access to essential healthcare and basic income security against sickness.^4^ An international UHC day since 2012 has popularised the benefits of universalism to a global audience; while most recently, UHC has been established as a target under the UN Sustainable Development Goals.

Such international agreements and movements highlight how UHC has returned with a vengeance to the international agenda, only a few decades after this ambition appeared ineluctably beyond reach. In 1978, governments and nongovernmental organisations from around the world signed the International Declaration on Primary Health Care (PHC) in Alma-Ata, USSR. Expressed in the slogan, ‘Health for All by the Year 2000’, this advocated an approach to health that was universalistic and grounded in human rights. Healthcare was to be ‘practical, scientifically sound and socially acceptable’, and premised on the active engagement of local communities.^5^ Subsequently, however, international health organisations withdrew from this ambitious vision, falling back on selective approaches to healthcare that were more targeted and cost-effective, such as immunisation.^6^ Classical models of social security also came into disrepute, as concerns were raised about the financing of PHC and the inability of social security systems to expand to meet the needs of people in developing countries.

Why did UHC suddenly return to international focus? One factor was the recognition that the imposition of user fees was, according to the WHO’s former Director-General, Margaret Chan, ‘by far the greatest obstacle to progress.’^7^ Recommended by international donors since the 1980s as a way of raising resources for healthcare, evidence had accumulated that user fees were a major barrier to entry, ‘a locked gate that prevents access to health care for many who need it most.’^8^ As if personally embodying the paradigm shift, in 2012 de Ferranti, one of the foremost experts to recommend user fees as a World Bank economist in the 1980s, praised the growing momentum behind UHC while accusing out-of-pocket health expenditures of burdening ‘sick and needy people … with most of the health-care costs’.^9^ Considering his various reports over the 1980s, which criticised the over-reliance on publicly financed healthcare in many countries, de Ferranti was perhaps the most surprising proponent of the re-emerging focus on financial risk protection and access to healthcare as a human right.^10^ However, his change of heart illustrates how various questions that had been marginalised in international discourse, such as how to deliver ‘health for all’ sustainably and equitably, were now firmly back on the table.

The recent consensus around UHC is only the latest high-point in a long struggle to embed UHC in international development. It mirrors an earlier episode in international health politics, surrounding the ILO’s 1944 Philadelphia Declaration and Medical Care Recommendation.^11^ These encouraged governments to provide comprehensive medical care to ‘all members of the community, whether or not … gainfully occupied’, and outlined two distinct pathways to UHC: compulsory social health insurance (SHI), and general taxation.^12^ Following the Second World War, however, this ambitious vision was defeated by an organised coalition of interests, including medical professionals, employers, and various governments. Against the political backdrop of the Cold War, these interests argued that comprehensive medical care equated to ‘socialised medicine’, and that poorer countries were insufficiently developed to extend healthcare to all. Wishing to develop a convention that all States could ratify, the ILO severely diluted its proposals: The 1952 Social Security (Minimum Standards) Convention presented an anaemic vision of the Philadelphia goals, permitting voluntary health insurance and a variety of exceptions and exclusions for low-income countries.^13^

This article explores the ILO’s approach to healthcare financing in the second half of the twentieth century. It shows that officials in the Organisation not only continued to promote health protection long after the original ‘universal dream’ had faded from view, but for a time, continued to be animated by its ideals, faithfully following the paths it laid out even if they could not be implemented immediately in the global South. The ILO’s approach developed in three main phases, which I have labelled ‘pragmatic gradualism’, ‘lost opportunities’ and ‘cooperative pluralism’.

In Latin America, South-East Asia and Africa, the ILO originally pursued a policy of ‘pragmatic gradualism’, establishing SHI schemes for smaller areas and subgroups of the population (often formal-sector employees) pending their extension to cover the whole country or population when conditions allowed. I will argue how the end of colonial rule in many countries permitted the ILO to expand its sphere of influence; however, its approach embodied questionable assumptions, such as that models of social security prevalent in wealthier countries could be straightforwardly rolled out given time and economic development.^14^ Political sensitivities around the status of health protection within the UN further undermined a coherent international approach to UHC.

The late 1960s and 1970s were a lost opportunity for the ILO, as the Organisation struggled to capitalise on new international agendas, such as PHC, to promote a coherent international strategy on UHC that married a concern about the optimal organisation and delivery of healthcare with its sustainable and equitable financing. I will show that while there was some limited collaboration between the ILO and WHO during this period, a more systematic and fruitful collaboration failed to materialise. This undermined the success of PHC and ultimately, the sustainable development of national health systems.

From the late 1970s, the ILO reacted to changing political winds, notably the ascendancy of the World Bank in global health, the rise of neoliberal ideology, and the forces of globalisation. These challenged the ILO’s traditional approach to health protection.^15^ I will highlight how in this new, more hostile climate, the ILO had to fight to maintain its legitimacy. ILO officials defended SHI as part of a pluralistic mix of measures, recognising that, while it could not be expanded to meet the total health needs of populations, it remained ‘the most reliable financing mechanism with the biggest financial potential.’^16^

## 2. The ILO and healthcare financing

The ILO may not be the first international organisation that comes to mind in relation to health, but its constitution has long embodied a commitment to shield people from the financial costs of illness. Established in 1919 following the Treaty of Versailles, the ILO was founded as a Western response to Bolshevism, its tripartite governance structure bringing together representatives of labour, employers and governments to forge international agreement on contentious issues of social justice.^17^ Initially concerned with workers’ rights and conditions, the scope of the ILO’s activity expanded by the Second World War to address comprehensive ‘social security’—the protection of entire populations against a range of social and economic risks, including the financial consequences of illness.^18^ The ILO became part of the UN in 1946. Based in Geneva, Switzerland, its work includes providing technical assistance to governments, conducting research, and promulgating international standards. These take the form of conventions, which are legally binding on member States, and recommendations, which are non-binding but provide a normative direction of travel. The organs of the ILO include a Governing Body composed of representatives of member States (including governments, employers and workers), a tripartite International Labour Conference (ILC) at which conventions and recommendations are agreed, as well as a permanent secretariat based in Geneva, the International Labour Office (for simplicity, I refer to the ILO and its Office together as the ILO). This article focuses on technical assistance, highlighting the significant advice and expertise ILO officials (as well as consultants in its employ) have offered to countries in the area of healthcare financing. While the ILO’s work in various countries is described, however, the article does not offer a detailed account of national cases. This gives it a somewhat technocratic slant, largely focusing on intellectual and policy developments in Geneva and in particular, the role of officials in the ILO Social Security Department, such as the directors Giovanni Tamburi and Michael Cichon. Nevertheless, it illuminates the broad changes that occurred to the ILO’s social health protection strategy and the Organisation’s position vis-à-vis other bodies, in particular the WHO.

In order to encourage the USA to ratify its constitution in 1946, and to avoid a repeat of the pre-war period, when it had refused to join the League of Nations, the WHO avoided direct engagement with the subject of healthcare financing, restricted to a ‘study and report’ role.^19^ This left the ILO as the de facto UN agency with power to directly advise governments on health financing, albeit solely in relation to social security. From the outset, this led to some friction with the WHO, which had direct responsibility for international health. While the ILO has never claimed to have specific medical expertise, for much of the period under discussion, the ILO’s expertise in health financing was underacknowledged and there was little formal liaison between the two organisations.^20^ They viewed each other with suspicion and resentment, an attitude that only began to ameliorate following the entry of the World Bank into international health in the 1970s and the beginning of structural adjustment programmes.

Recent histories of the ILO have stressed that while geopolitical trends such as the Cold War and ideological movements such as neoliberalism have constrained the ILO’s capacity to promote social justice, ILO officials have exerted considerable influence over international policy, working in complex transnational networks that transcended national political domains.^21^ They were not simply agents of particular governments, though particular groups, such as US New Deal experts, dominated discussions at various points in time, and particular national models of healthcare, such as the British National Health Service (NHS), were also influential periodically. Corroborating these histories, this article will demonstrate that while the ILO has recently faced challenges to its authority, its officials have nevertheless made a meaningful contribution to the improvement of health security internationally.^22^ The ILO has been adept at repositioning itself in response to global political trends, and taking advantage of such movements to make a continued case for social justice and its own political relevance.^23^ Indeed, political pragmatism has been a defining characteristic of the ILO ever since its inception, punctuated only rarely by moments, such as the 1944 Philadelphia Declaration, when idealism won the day. I begin by discussing this early optimistic model of UHC, highlighting the specific approaches to health protection it embodied, and the reasons for its weakening at the 1952 ILC.

## 3. Mid-century models of UHC in the ILO

As outlined above, the 1944 Philadelphia Declaration and Medical Care Recommendation endorsed two distinct approaches to UHC: compulsory SHI and general taxation. Members of the ILO Social Security Department such as the economist Laura Bodmer played an active role in fleshing out this early vision of UHC, going far beyond their role as ostensibly ‘neutral’ technical experts. They were inspired by recent developments in Britain, notably the 1942 publication of the Beveridge Report, which established the blueprint for Britain’s post-war welfare state (the officials Maurice Stack and Osvald Stein had given evidence to the Beveridge Committee); American New Deal progressives such as I.S. Falk were also influential in shaping the ILO’s thinking, as was the establishment in 1938 of the New Zealand NHS. The historical influence of German social insurance experts in the ILO, as well as the importance of contributory social insurance in many European countries, explains why SHI was chosen as a template for UHC alongside British-style general taxation.^24^ SHI has numerous organisational variants, and the concept had evolved since its first implementation in Germany in 1883.^25^ Broadly, it refers to contributions by salaried employees and employers to third-party funds which act as the primary payer/purchaser of healthcare; often compulsory, it differs from general taxation largely on the basis that healthcare is financed directly through earmarked contributions or premiums, rather than indirectly through taxes levied on the population (in some variants, such as national health insurance in Britain before 1948, the government also pays contributions).^26^ Considerable differences between systems exist in terms of the number of funds, their benefits, governance and membership, the degree of government oversight, and whether individuals can exercise choice.^27^ From the ILO’s perspective, however, general taxation and compulsory SHI were thought to have the same effect, namely that once fully developed, they permitted population-wide access to ‘comprehensive medical care’ and income security from illness. In the case of SHI, the contributions of groups who could not pay, such as the ‘indigent’, would be paid for by public financing, thus the salaried population would subsidise the care of the non-salaried (the redistribution principle).^28^ In the interwar period (1919–1939), concerned with the financial stability of sickness funds, ILO officials had supported the expansion of compulsory SHI in Europe.^29^ Now, SHI was viewed as a basis to expand health coverage elsewhere, and in practice was the focus of their efforts.^30^

As a more holistic conception of ‘social security’ crystallised in the mid-twentieth century, the 1952 Social Security (Minimum Standards) Convention sought to codify developments across several branches of the ILO’s activity, including maternity benefit, old-age pensions and sickness insurance. Attempting to establish a minimum bedrock of social standards, the weakening of medical care and sickness benefit came about due to fierce opposition by organised interests from both industrialised and developing countries. For example, the recently established World Medical Association lobbied intensely against the ILO’s proposals, arguing that they interfered with the privileged relationship between doctor and patient. Industrialised countries with a strong tradition of voluntary health insurance, such as the USA, resisted any suggestion of compulsion, dismissing it as ‘socialised medicine’, while developing countries such as India argued that they were insufficiently developed to offer universal health care. Imperial powers such as Britain also feared the consequences of social security for their colonial dominions, even while the Beveridge Report promised universal welfare for its own citizens.^31^ This opposition highlights that health systems financing was never purely ‘technical’, of esoteric concern only to health administrators, economists or international officials. Rather, it raised complex questions about how healthcare should be organised in countries, and who should pay for it, rubbing against the vested interests of groups keen to preserve the status quo.

In the end, the political idealism that underpinned the 1944 Philadelphia Declaration ebbed away. In particular, voluntary health insurance was preserved as an acceptable approach to health coverage, undermining much of the intention behind the Medical Care Recommendation. Moreover, while the 1952 Convention had been drafted with the hope of allowing as many developing countries as possible to ratify, very few ultimately did. As the opposition from countries such as India revealed, countries emerging from colonisation were hesitant to commit themselves to a new international instrument. The presence of large informal sectors in much of the developing world explains in large part this reluctance: the 1952 Convention was premised on organised labour relations and formal economies, and thus implicitly embodied the experiences and interests of the global North.^32^ An early opportunity to embed UHC in international development was thus lost—yet, ILO officials continued to hold onto these ideals, even as the wider international community was not ready to commit.

## 4. Pragmatic gradualism, 1952–1959

While the 1952 ILC was a setback for ILO idealists, in some respects the postwar years were business as usual. The ILO attempted to adapt the ‘classical’ models of social security for application in developing countries, meeting with little success.^33^ The end of colonial rule in many countries presented significant opportunities for the ILO to expand its sphere of influence. Between 1950 and 1965, the ILO dispatched around 3000 expert missions to 100 countries.^34^ This represented a major gear-change: hitherto, technical assistance had formed only a small part of its activities. Forming part of the UN’s Expanded Programme of Technical Assistance after 1949, ILO assistance was designed to transfer knowledge from the global North to the global South. The systematic promotion of social security in Africa was delayed by the deliberate obfuscation of the colonial powers who resisted the ILO’s ‘universalistic model of development’, establishing a parallel agency, the Combined Commission for Technical Co-operation in Africa, in part to limit the ILO’s reach.^35^

Despite the lacklustre response to the 1952 Convention, many postcolonial governments were receptive to the ILO’s universalist agenda. Inheriting health systems from their former colonisers, many expressed their intention to provide healthcare that was nominally free to access for all their citizens. Engaging with ILO technical assistance (even without ratifying any conventions) provided a powerful signal of their political legitimacy.^36^ ILO missions to countries such as Senegal, Gabon and Mali attempted to transpose Western bureaucratic models, undertaking actuarial analyses, surveys of business, and reviews of legislation to support the extension of SHI.^37^ In Gabon, which achieved independence from France in 1960, one mission led by the ILO’s Guy Perrin even went so far as to recommend the progressive establishment of an NHS, considering the administrative costs associated with French-style social insurance. Gabon’s existing system of health protection consisted of a scheme for salaried employees and their families, and an underdeveloped public scheme, which was generally free to access, with various charges levied at hospitals. Gabon’s level of economic development precluded the immediate establishment of an NHS, thus, until economic conditions improved, Perrin recommended that various steps could be taken by the government to improve access, such as eliminating charges levied by doctors.^38^ The underlying idea was that given time and orderly development, health protection could be gradually extended to the whole population. This stance was supported by dominant theories of development, which assumed that with the expansion of the formal workforce, ever larger segments of the population would fall under social security as it was traditionally conceived.^39^ As I have already suggested, this assumption embodied various neo-colonial assumptions about development, implicitly taking as its template the evolution of social security in Western Europe. Thus, the dominant model of development yoked low-income countries to a model of social security conceived and operationalised in a context far removed from the one where it was now being transplanted. In Latin America, which was another focus of ILO activity, officials declared that national schemes of social insurance were ‘beyond the resources of most countries’, and that their hasty introduction could lead to ‘legal sham and administrative chaos’. The gradual extension of social insurance, by contrast, was ‘wise and realistic’.^40^ The statutes of a number of Latin American countries implicitly embodied a gradualist approach, envisaging the progressive extension of sickness benefit and medical care, as economic conditions improved, to new territories and classes of the population as a social right.^41^

In the late 1960s, the gradualist approach was given further legal substance when the ILO revised its 1920s conventions on sickness insurance in industry and agriculture. The 1969 Medical Care and Sickness Benefits Convention introduced higher standards for these branches of social security than the minimum standards established in 1952, stipulating wider coverage, access to more services, and higher levels and longer duration of sickness benefit.^42^ An accompanying recommendation went even further, capturing some of the intent, if not the idealism, of the 1944 Medical Care Recommendation. It envisaged the progressive extension of health protection ‘by stages if necessary, and under appropriate conditions’ to casual workers, dependents, all economically active persons, and eventually, to all residents.^43^ The 1969 instruments were drafted at the behest of the ILO’s Committee of Social Security Experts, who pointed out that the pre-war conventions had become ‘obsolete’ in light of social security developments over the previous forty years.^44^ Although they complemented and exceeded the standards in the 1952 Convention, they were not intended as the associated ‘advanced standards’, a proposal that had been raised ahead of the 1952 ILC, but ultimately dropped.^45^ For the ILO, the 1969 instruments demonstrated its continued commitment to UHC as the final destination of health protection, even when the exact path in countries remained unclear. They provided a normative direction of travel for countries to follow, even if they could not be implemented straight away. Unsurprisingly, however, few countries were willing to embrace the higher standards: as of 2019, only 16 countries have ratified the 1969 Convention.^46^

One would expect, with this effort to promote health protection worldwide, that the ILO would have found a natural ally in the WHO. If anything, the opposite was the case: the WHO’s inauguration in 1946 caused serious tensions with ILO. Institutional rivalry between the ILO and the leading international health organisation was not a new phenomenon: the ILO’s expertise in the organisation of medical care under sickness insurance schemes, for example, had led to tension with the WHO’s predecessor, the League of Nations Health Organisation.^47^ However, the establishment of the UN and the commencement of the Cold War brought a new political dynamic to this competition. The potential for functional overlap was one obvious source of strain, each organisation being keen to maximise its area of responsibility, and thus technical authority; another was the divergence of their approaches to healthcare in the post-war period, with the WHO more exposed to the conservative, anti-social-security rhetoric that emanated from the USA. This is evident from the WHO’s hostile reaction to the report of the Commission on Organisation of Medical Care (COMC) in 1952.^48^ Initiated in response to the ILO’s draft social security convention, the COMC featured prominent interwar advocates of social medicine such as Henry Sigerist and René Sand. The Commission advanced proposals, which were anathema to organised interests such as the American Medical Association, such as universal healthcare, salaried medical services and group practice. The report was roundly attacked and promptly disowned by the WHO’s Executive Board; it was only sent to the ILO on the understanding it should not be considered formal WHO policy; and Milton Roemer, Sigerist’s protégé and head of the WHO’s Occupational and Social Health Section, was forced to resign.^49^ Henceforth, the WHO avoided the contentious issue of social security in its fieldwork in countries, although it conducted research on its medical and financial aspects.

The consequences of the WHO’s retreat from this space were two-fold. Firstly, as explained above, the ILO was left as the only international organisation with power to directly advise governments on healthcare financing and organisation. Secondly, relations between the two organisations in the 1950s and 1960s in relation to medical care were largely restricted to research, even as the demands of health systems development arguably necessitated a more direct approach. One such area of joint research in this period was understanding the rising costs of healthcare internationally—how the growing demand for health services, as well as the rising price of inputs such as pharmaceuticals and medical equipment, were placing increasing pressure on health financing systems, including in the global North.^50^ The ILO’s command of healthcare financing and its close grasp of events at national level through its relationship with the International Social Security Association, an international forum for social security agencies, explains why it was the first international organisation to respond seriously to these concerns, initiating a study as early as 1953.^51^ From 1956, however, WHO was also drawn into the debate, alarm having been raised by a Chilean consultant, Hernán Romero. Brian Abel-Smith, Lecturer in Social Administration at the London School of Economics and Political Science, was subsequently invited to Geneva to advise the WHO on a comparative methodology for international health accounting, but sensing that it had limited expertise in this area, the ILO was also called on for advice.^52^ The result was a joint UN/ILO/WHO study group in July 1958, bringing together participants such as Abel-Smith, Milton Roemer, and Laura Bodmer of the ILO Social Security Department. This culminated in a series of ILO and WHO reports which provided the foundations for the cross-national study of health expenditure: In 1959, the ILO published *The Cost of Medical Care*, Bodmer’s examination of medical care expenditures in thirteen social security systems; this was followed in 1963 by Abel-Smith’s WHO report, *Paying for Health Services*, which examined health spending in six countries.^53^

In relation to healthcare organisation, the ILO had significant expertise to offer the WHO, especially since health services were provided in many countries under the ambit of social security. However, the ILO was involved only marginally in the WHO’s earliest deliberations on the subject, dispatching representatives to the Expert Committee on the Organisation of Medical Care in the late 1950s.^54^ ILO expertise was entirely absent from the more extensive work of the WHO Expert Committee on Public Health Administration throughout the 1950s and 1960s, and more disconcertingly, two enquiries in the late 1960s on health planning. As countries sought to develop rational approaches to the financing and delivery of healthcare, based on the actual health needs of populations, this exclusion only served to undermine coherent international thinking on the topic, and ultimately, the success of national health planning programmes. The WHO’s ‘study and report’ role, as discussed above, meant that the agency could not involve itself in normative questions about how countries should fund healthcare, and thus it was largely considered from the standpoint of information: as an input to the health planning process alongside other resources such as medical personnel and drugs.

The ILO’s studies on healthcare organisation in the 1960s were thus almost entirely divorced from WHO’s: it was the global spread of social security programmes, not health planning, that motivated ILO to launch a study programme on medical care organisation under social security in 1966. The study’s aim was to examine the costs and benefits of different social security models and their effects on healthcare. Eight countries were chosen for detailed study based on their supposed representativeness ‘of the most specific and important types of medical care under social security’: Belgium, Canada, Ecuador, the Federal Republic of Germany, India, Poland, Tunisia and the UK.^55^ The final report, written by Roemer (now at the University of California in Los Angeles), described in detail the historical evolution of medical care and the various arrangements under which it was financed and provided. UHC was considered particularly efficient from an administrative perspective, negating the need for complex eligibility criteria and qualifying conditions. It was also portrayed as the natural culmination of long-term international trends. Unified national health services, such as in Britain, were considered to be the logical endpoint of this process, beyond a threshold when it became ‘administratively easier to combine organised efforts under a co-ordinated or unified authority’.^56^ Roemer’s conclusions were not particularly surprising considering his earlier role on the COMC, but the report demonstrates how the ILO, together with academic champions of social security such as Roemer, continued to advocate for UHC despite its wider international marginalisation.

In sum, after 1952 ILO officials continued to be animated by the universalist ideals of the 1944 Philadelphia Declaration. However, their activities in the global South were hamstrung. Not only did political and economic impediments stand in the way of countries embracing UHC, but the ILO was committed to specific models of health protection inherited from the global North, whose applicability in poorer countries was unclear. More damagingly, Cold War politics prevented a coherent and cooperative approach to health protection developing in the UN. Nonetheless, the ILO’s work embodied a certain pragmatism that allowed it to continue the push for UHC, regardless of these difficulties.

## 5. Lost opportunities, 1969–1978

In 1969, the ILO won the Nobel Peace Prize for its promotion of social justice, an event that some commentators have taken to be the ‘zenith’ of the ILO’s international influence.^57^ Unfortunately, this article does little to diminish this interpretation. From the 1970s, the ILO increasingly found itself having to adapt to outside influences, notably the ascendance of the World Bank in international health, and the growing influence of the Organisation of Economic Co-operation and Development (OECD) over social policy in the global North.^58^ The global reach of the ILO was also damaged by the temporary withdrawal of the USA from the Organisation’s membership along with the financial contributions that bolstered its pocket-book. With this loss of global influence and prestige, the earlier ideological vision that drove the ILO’s promotion of UHC further evaporated. However, it was still present among some of its officials, who attempted to leverage new global movements to support the extension of SHI.

One such movement was the international drive to plan and expand healthcare in low-income countries, which, finally, prompted formal collaboration between the ILO and WHO. Generating urgent questions about optimum methods of financing and delivering healthcare, health planning naturally called for close coordination between the work of the two organisations, but as noted, nothing resulted in the 1960s. With the start of the new decade came recognition that some coordination was essential, with a series of rudimentary attempts to bring their budgets and work programmes into line. Correspondence between the ILO and WHO, such as its Director of Health Services Strengthening, Kenneth Newell, reveal that WHO officials accepted that its record on health services financing had been ‘poor’ and that its work had been limited by the ‘highly political aspects … involved.’^59^ The softening of the political situation increasingly permitted the WHO to work with colleagues elsewhere.

The first formal area of ILO-WHO collaboration outside occupational safety and health was the Joint Committee on Personal Health Care and Social Security in 1970. Chaired by Ricardo Asturias Valenzuela, the former Minister of Public Health for Guatemala, the Joint Committee explored the relationship between social security and healthcare services around the world, noting the general trend for the ‘gradual extension of coverage’.^60^ The Joint Committee did not emphasise UHC, which was considered a distant prospect for many countries; instead, emphasis was placed on closer coordination between different parts of health systems, and greater attention to efficiency and cost. The imperative for all countries was to efficiently ‘utilize given foundations, of whatever type they may be’ and to ‘achieve co-ordination in the operation or delivery of personal health services … even if financial support is derived from multiple sources.’^61^ SHI was positioned as a stable and powerful instrument for countries to expand healthcare to particular population groups. However, authorities needed to carefully consider the impacts of such schemes, their political acceptability, and whether they increased resources for the health sector, or as suspected by some critics, diverted resources away from public health ministries.^62^ (Professional connections between the ILO Social Security Department and academic champions were once again pivotal to dispel such assumptions: in an exchange of correspondence in the early 1970s, the Director of the Social Security Department, Giovanni Tamburi congratulated Roemer on a recent article which challenged the belief of some health planners that social security financing was ‘incompatible’ with public health provision).^63^ Despite the cautious tone of the Joint Committee, Tamburi was pleased with the result, considering that the meeting had helped ‘reassert the competence of the ILO’ in relation to the financing of health care under social security.^64^

The WHO, for its part, was noncommittal regarding the optimal route to UHC. Barred from promoting particular models, its approach is best described as naturally pluralistic: whatever worked, depending on the situation of the country in question. By the mid 1970s, its thinking on health services, alongside UNICEF, had consolidated around the promotion of ‘basic health services’, such as maternal and child healthcare. This was prompted in part by the growing political strength of newly independent nations in Africa and Asia, which called for the establishment of a new international economic order; it was also encouraged by the diffusion of lessons derived from developing countries, such as the Chinese barefoot doctor programme.^65^ Recovering from the failure of national health planning in the 1960s, as well as global malaria eradication, the message emerging from this movement was that a new approach to planning was needed based on the economic and social realities of developing countries. Methodologies that were overly formal, or relied upon massive transfers of technology and expertise from developed to developing countries, were to be discouraged. Elaborating their thinking on PHC in a report ahead of the 1978 conference in Alma-Ata, USSR, the Directors of WHO and UNICEF criticised the unquestioning embrace of social security in developing countries, including systems based on general taxation. For them, the automatic adoption of financing mechanisms that were prevalent in the global North was equally as ill-advised as the use of inappropriate technology. An ‘open mind’ to financing was instead needed, and SHI was presented as just one of many routes towards ‘health for all’, as indicated in the conference’s recommendations.^66^

All this, of course, had profound implications for the ILO, not only because the ILO was committed to specific pathways to UHC, but also because its technical assistance was predicated on the very transfers of tools and expertise that were now being questioned. PHC also presented a further, more significant challenge to the ILO’s authority: it exposed the huge gulf between the aim of social security, to provide comprehensive protection for entire populations against socioeconomic risks, and the reality in many countries, namely, the adoption of piecemeal insurance schemes for specific risks and smaller segments of the population, often those in the formal sector such as civil servants.^67^ Pragmatic gradualism, for all its supposed prudence, had failed to extend health protection quickly enough to those populations most in need.

Considering that it threatened to undermine the ILO’s work on health protection, it is not surprising that the ILO’s reaction to PHC was distinctly ambivalent. On one level, some ILO officials declared their ‘great interest’, pointing out how the drive to establish basic health services was compatible with the ILO’s own agenda to promote ‘basic needs’.^68^ This agenda had evolved out of the World Employment Programme (WEP) initiated by Director-General David Morse in 1969. ILO missions to countries such as the Philippines observed that economic development along conventional lines had done little to generate employment and increase the prosperity of the global poor. Forming part of a broad critique of traditional theories of economic development by figures such as Gunnar Myrdal, the ‘basic needs’ agenda posited that poverty, rather than unemployment per se, was primarily to blame for underdevelopment. In his 1968 book *Asian Drama*, Myrdal highlighted that the concept of ‘employment’ was only loosely applicable in developing countries, where informal, rather than formal employment, tended to dominate; the pressing issue was instead ‘labour utilisation’, which was limited by human factors such as education and health.^69^ As per the ILO’s overall focus on work, the WEP advanced an employment-centric agenda that emphasised youth training, international trade and rural development as a way of reducing poverty. The basic needs approach reached its apotheosis at the 1976 World Employment Conference, which advocated a fundamental reorientation of international development towards alleviating poverty. At the conference, basic needs were defined broadly to include food, shelter, sanitation, safe drinking water, education, and health (the latter dovetailing with the work of WHO). Unfortunately, despite the adoption of an ambitious declaration and programme of action, little became of this agenda within the ILO.^70^ Little was done to explore the necessary mechanisms of change, leaving it to individual countries to search blindly for a way forward. On the issue of health, the question of how developing countries should provide and finance healthcare was left unanswered. While the agenda lost momentum within the ILO, other institutions, notably the World Bank, became increasingly interested in basic needs in the late 1970s. Under the presidency of Robert McNamara (1968–1981), the former US defence secretary, the concept of basic needs underpinned its own growing involvement in international health.^71^

Other ILO officials, however, were clearly disengaged with PHC, for the ILO initially declined an invitation to address the 1978 Alma-Ata Conference, believing that the receipt of relevant documents was sufficient. Neither the ILO’s Director-General, Francis Blanchard, nor Giovanni Tamburi were consulted about this decision, which illustrates how the International Labour Office was far from unified. Work in the Office was atomistic, with departments often failing to consult each other, and while PHC had obvious relevance for the work of the Social Security Department, it was far removed from the work of others. In general, healthcare was at the periphery of the ILO’s focus—thus, the Alma-Ata Conference could be casually dismissed, despite its wider implications for ILO standard-setting and technical assistance.

In any case, the initial mistake was soon rectified, and the ILO agreed to attend, its senior officials cognisant of the political capital attendance would bring. For Jacques Lemoine of the ILO’s International Liaison Office, ILO participation was crucial: ‘[PHC] is an important element of the basic needs approach including popular participation. We must broaden our horizons beyond income redistribution, and work with others.’^72^ A note of caution, however, was also expressed by Tamburi, who evoked the familiar themes of suspicion and resentment of its neighbour across the Avenue Appia:

PHC is provided in many developing countries by social security programmes and the ILO has been instrumental in the planning and organisation of such health services.… WHO has shown a tendency to underestimate and even to oppose the development of health services through social security, an attitude which many developing countries have criticised and rejected.^73^,^73^,^73^

All this suggests the keen sense of threat the ILO continued to feel from the WHO and its PHC agenda. The solution proposed by some officials, such as the consultant Derick Fulcher, was to leverage the new agenda in support of the ILO’s aims. They argued that PHC could be pursued through better coordination between medical care organisations, improved health education and training, as well as the ‘direction of social security resources towards national PHC’.^74^ They also claimed that PHC increased the viability of SHI as a financing mechanism. The need to find new financial solutions for healthcare expansion became particularly acute after 1978, when concerns were raised about the feasibility of ‘health for all’. A report from the WHO itself noted a deficit of some $50 billion a year between existing health expenditures in developing countries and that required to achieve UHC.^75^ This raised alarm bells, and within a year, opponents of comprehensive PHC were beginning to promote a more targeted package of cost-effective interventions, such as oral rehydration and breast feeding.^76^ In addition, mounting sovereign debts in the early 1980s, and the imposition of structural adjustment measures by the International Monetary Fund (IMF) and World Bank, meant that public health expenditures were severely curtailed in many countries. SHI thus became an attractive extra-budgetary solution for governments to fund healthcare, an ILO report in 1990 noting the ‘increased awareness of the place that compulsory health insurance, although limited in its population coverage, occupies or should take within a national health policy.’^77^

It is not surprising, therefore, that despite reservations about its wider impact on the Organisation, ILO officials such as Surendra Jain, its Assistant Director-General, expressed their commitment to PHC.^78^ In the early 1980s, the ILO cooperated with the Pan American Health Organisation (PAHO) to consider the implications of social security for PHC in Latin America, holding regional consultations in Mexico and Colombia.^79^ A PAHO Resolution in 1981 repeated the assertions of the 1970 Joint Committee, establishing that the path to more efficient healthcare lay in improved coordination between social security and public health institutions.^80^ For Tamburi, who was present at these consultations, ‘there was no hope that generalized “access” to health or a correction of the present “distortions” in the system could be attained in the short run.’^81^ However, ‘proceeding by slow and gradual stages’, health protection could eventually be secured for the majority of Latin American citizens.^82^

From the late 1960s onwards, there were thus numerous opportunities for the ILO to promote its vision of health protection internationally. There is evidence that ILO officials attempted to seize these moments when they became available. However, in the absence of a more fruitful ILO-WHO collaboration, this engagement with contemporary development issues could only ever be superficial, and a coherent international strategy on health protection embodying a substantive concern with healthcare financing failed to materialise.

## 6. Co-operative pluralism, 1978–2012

If PHC prompted the ILO to reappraise the role of social security in financing healthcare, then from the late 1970s it was forced to respond to a more concerted attack on its authority—on two fronts. In the global North, rapidly increasing social expenditures, including healthcare, attracted the attention of the OECD, an international forum for industrialised countries. Adopting an expanded social policy remit, as well as a distinctly free-market ideology by 1980, the OECD attacked the seemingly ever-growing costs of social security in the West amid widespread unemployment and reduced economic growth.^83^ An OECD conference in Paris even went so far as to pronounce a ‘crisis’ in post-war welfare states, prompting the ILO to convene a group of social security advocates including Brian Abel-Smith to rally a robust defence.^84^ OECD reports over the 1980s and 1990s stressed the need for efficiency and cost-effectiveness in healthcare, as well as mechanisms to control cost, such as budgetary caps, patient co-payments and competition.^85^

In the global South, the World Bank began to lend directly to the health sector for the first time. Also channelling neoliberal logic, from the mid 1980s the Bank advocated the introduction of user charges at the point of service. According to the Bank’s economist, David de Ferranti, ‘the conventional and still growing faith that health care should be totally paid for and administered by government needs to be vigorously challenged’.^86^ ILO officials resisted, considering user charges ‘difficult to implement’ and ‘highly questionable’, but were powerless to stop their widespread implementation, often as a prerequisite for continued development lending and debt relief.^87^ The group of social security advocates convened in 1984 was also incensed, believing that the use of co-payments equated to ‘de-insurance’.^88^

Despite this defence, by the early 1990s the ILO increasingly accepted a ‘pluralistic’ model of healthcare financing in low-income countries: an ‘integrated, coordinated overall health care financing system which consists of different financing subsystems with clearly defined scopes and mandates.’^89^ The problem, as outlined by Michael Cichon, actuary and later Director of the Social Security Department, was that while SHI schemes were ‘fair and equitable’ for covered populations, and allowed individuals to contribute to the costs of healthcare, they could only form a small part of the overall arrangements for healthcare in developing countries for the foreseeable future:

While social security can directly or indirectly subsidise health care delivery systems for uncovered persons, it cannot alone (and this is the root of all its problems) provide and finance comprehensive care for all the total population in developing countries with a relatively small formal sector. The resulting contribution burden for formal sector employees and employers would simply be too big and the collection of contributions from the informal sector does seem to meet with often insurmountable administrative obstacles.^90^,^90^

Elsewhere, Cichon and the Director of the Social Security Department, Colin Gillion, noted that pluralistic schemes were ‘unavoidable’ and ‘inevitable’.^91^ Gillion was especially frank in a report on healthcare in Africa published in 1993: ‘It appears that the times of the grand designs for health care systems based on economic theory or philosophical and political convictions are over. … [D]ifferent forms of organization and financing of health care can, and most likely will have to, co-exist in one country.’^92^ Far from being the one-way road to comprehensive medical care it had once been, the ILO now considered social security just ‘one of the more efficient among a limited number of tools in the toolbox of health policy-makers’.^93^

The promotion of pluralism was not without problems. As Cichon recognised, ‘all financing subsystems including the social security (sic) have inevitably some negative side effects.’^94^ On the one hand, the social security system needed to provide ‘differentially better care’ for the covered population to be politically accepted. On the other hand, there was the ‘societal obligation’ for social security to subsidise the care of the non-covered population.^95^ Pluralism accepted that social insurance systems often created islands of privilege, with covered groups reluctant to expand their benefits to others, yet it provided no easy solutions: ‘Delicate and imaginative compromises will have to be found …’.^96^ The ILO’s overall intention was to avoid the creation of ‘isolated autonomous subsystems … The pragmatic “bottom policy line” is one can accept more inequity as long as after the introduction of a social security scheme all individuals in a given country are better off than before.’^97^

Given this risk, why did the ILO, which had steadfastly supported the extension of SHI (and to a lesser extent, single-payer or NHS models), now assume this apparently fatalistic position? There are at least three possible answers. The first is that the thinking of ILO officials was increasingly dominated by the neoliberal logic of health economics emanating from the World Bank and OECD. Accepting the limitations of public-sector involvement in healthcare, while emphasising the benefits of a public/private mix of services, the language was disseminated worldwide through informal contacts between international officials, inter-agency meetings, and international conferences on health economics that officials attended, such as the First European Conference on Health Economics in Barcelona in 1981.^98^ A second answer is that the ILO was simply facing up to political reality. Outlining what he termed the ‘social insurance dilemma’, Cichon accepted the long-established fact that SHI in poorer countries tended to favour better care for a small minority of people at the expense of adequate care for the entire population. While SHI potentially freed up funds that governments could target at the poorest in society, there was no guarantee they would actually do so.^99^ Further, with the collapse of the Soviet Union and the ascendancy of neoliberalism as the guiding ideology of international development, the ILO had little choice but to accept that many countries were moving towards more decentralised health systems, which embraced the free market.^100^

A third answer is that pluralism was a pragmatic way of defending the role of SHI in a heterogenous system of healthcare, and thus served to reinforce the ILO’s legitimacy in a hostile political climate. For example, as Cichon wrote in 1991, ILO attendance at health economics meetings meant that there was ‘an ongoing debate about the potential of health [insurance].’^101^ Pluralism sought to integrate SHI closely into an overall national strategy for healthcare, and as formulated by Cichon, explicitly permitted the contracting of services and potentially, the development of an internal market as recently introduced in the British NHS. Thus, while the ILO’s field of action was constrained by the new policy environment dominated by the World Bank and OECD, SHI was preserved as a policy option for financing and delivering healthcare: ‘There is certainly no defined universal solution for all health care financing problems,’ Cichon wrote ahead of a meeting at the World Bank in 1992, 'but there might be a framework for such solutions which involve a strong social insurance component.’^102^ Pluralism was a way for the ILO to remain politically relevant, and to keep its place at the international table.

The ILO was not alone in this aim. Its closest ally was, ironically, the organisation it had been most begrudging towards: the WHO. As early as 1984 there was agreement that ‘it was essential for the “social” specialised agencies to present viable alternatives to the IMF and World Bank’s stringent adjustment policies’, and officials in both organisations realised that their best hope lay in joining forces.^103^ ILO-WHO collaboration intensified in the late 1980s, facilitated by personal contacts between members of the ILO Social Security Department and the WHO’s Strengthening of Health Services Division, such as Eleuther Tarimo, Andrew Creese and Bill Newbrander. To formalise this cooperation, in 1991 they drafted a joint statement on strategies to improve the economic and financial basis of health systems. This acknowledged that health systems in developing and transition countries were necessarily pluralistic and that ‘in spite of all efforts, adequate health care for all has not yet been achieved.’^104^ By the early 1990s, therefore, there is evidence of a thawing of ILO-WHO relations. For Cichon, collaboration allowed the organisations to remain ‘competitive’ with the World Bank, and to increase their ‘mutual political power base’.^105^ Additionally, co-operation was thought to increase the organisations’ mutual technical capacity, since both the ILO and WHO had ‘understaffed and underfunded health financing units’.^106^ Co-operation soon began to bear fruit, with the 1994 publication of a guidebook on SHI for use by health planners.^107^

The incubator and laboratory for the ILO’s pluralistic approach was central and eastern Europe (CEE). Following the end of communist rule, CEE countries sought to (re)introduce SHI as part of a decentralised, pluralistic model of healthcare. Universalism remained a cornerstone of the both the former and new systems, enshrined in the laws and constitutions of countries such as Poland.^108^ However, a fundamental problem was that the heavily centralised, communist model of healthcare was firmly engrained, and the transition to a marketised, pluralistic system exposed the population to numerous risks, such as rising costs, ‘wild uncontrolled’ privatisation, and reduction in healthcare quality.^109^ Economic risks, such as hyperinflation, and political instability also threatened an orderly transition, and interfered with policy development. The picture that emerges from ILO reports is one of a headlong rush by countries to embrace SHI, as well as caution on the part of the ILO; by 1992 it had become clear to Cichon that ‘the introduction of insurance financing is no universal panacea nor an immediate solution.’^110^ Nevertheless, the ILO attempted to bolster capacity in CEE countries, focusing on actuarial, budgeting and statistical capability, and policy analysis, such as advice on social security legislation. For example, in the Czech and Slovak Federal Republic (CSFR) ahead of its dissolution, an ILO mission in 1990 performed an actuarial evaluation which was integral to the country’s social security reforms. In 1991, ILO officials, along with members of the European statistical agency, Eurostat, provided assistance to CSFR officials in restructuring statistical systems necessary to monitor the transition process. In June 1992, the ILO held a summer school in SHI management for CEE officials at its training centre in Turin; and in September 1992 an inter-agency mission led by Gillion assisted the Polish government with the preparation of a social security white paper. All these activities point to the ILO’s active role in CEE health-sector reforms, going beyond mere project planning and monitoring. For the Organisation itself, these reforms presented an ideal opportunity for it to market its services, its reports clearly indicating that the Organisation expected that close cooperation with governments and other international agencies such as the World Bank would open up new opportunities in the region in future.^111^

Influenced in part by these developments, by the early 1990s ILO officials recognised the increasing demand for specialists to work on country projects, and the ‘enormous momentum’ behind the need for advisory services.^112^ As countries sought to ‘off-load’ healthcare financing, ILO officials were brought in as short-term research consultants for proposed SHI schemes in countries such as Zambia, Trinidad and Tobago, and Montserrat.^113^ In the early 1990s, ILO officials oversaw the implementation of national health insurance in Nigeria, providing actuarial analysis, support for project design, and the development of administrative infrastructure.^114^ However, one notable country which was a target for ILO assistance was Thailand, whose government was committed to extending health coverage. In the early 1990s, ILO consultants supported the planning and implementation of a new SHI scheme for private-sector employees, assisting with the design of its delivery and payment system. The success of this scheme subsequently enabled the Thai government to increase coverage to smaller enterprises, and as the scheme built up reserves over the decade, thought was given to extending coverage further, including to the informal sector.^115^ From 2001, the Thai government pledged to extend UHC to the entire population, announcing the ‘30 Baht’ Programme by which people could purchase healthcare through a small co-payment. Rapidly deployed throughout Thailand, UHC was achieved just a year later. This success has been credited to the political commitment of successive Thai governments, the long-term development of health infrastructure, ‘reformist bureaucrats’ in the Thai Ministry of Public Health, and, in particular, the political advocacy of groups such as the Thai Rural Doctors Society, which meant that even with the Asian financial crisis from 1997, the political will to promote UHC remained.^116^ However, the success of UHC in Thailand also depended on a pluralistic approach, whereby existing schemes were used as foundations to extend coverage to the whole population.^117^ ILO expertise was certainly not decisive in these developments, and UHC was likely achieved despite the ILO’s involvement.^118^ Even so, it is important to recognise the practical contribution of ILO officials, who, as Joseph Harris has observed, helped give the UHC program the imprimatur of scientific authority by approving is initial budget estimates. With strong links to the Rural Doctors Society, ILO officials helped ‘“immunize” the doctors’ estimates from criticism’ and offered support for the scheme when it faced administrative hurdles and political opposition. Doctors who opposed the scheme did not have such supportive international networks.^119^ While this support can be viewed as ‘symbolic’, ILO advice encouraged the Thai government to develop a system that embodied the Organisation’s key principles of equity and redistribution.^120^

The year 2001 also marked a new watershed in the ILO’s approach to health protection. Following an ILC directive, the ILO launched a Global Campaign on Social Security and Coverage for All to seek ways of extending coverage to groups that remained unprotected. Linked to the UN Millennium Development Goals, a commitment to ‘universal access to social health protection’ was a central component of the strategy developed. Building upon the ILO’s experience of health protection over the previous half century, as well as the success of UHC in Thailand, the strategy confirmed ‘the contribution of all existing forms of social health protection’ to UHC.^121^ Pluralism was now enshrined as the most effective route to UHC in low-income settings, effectively killing off any ideological commitment to particular pathways (such as single-payer models) that lingered in the ILO’s thinking. The social health protection strategy was connected to the ILO’s Decent Work Agenda, which formed the Organisation’s response to the challenges of globalisation and the attacks on its legitimacy from the institutions of global trade and finance, such as the World Bank and World Trade Organisation. This agenda was in turn linked to the poverty reduction strategies of the IMF and World Bank, highlighting how the ILO attempted to leverage its connections with the Bretton Woods Institutions to advance its goals. On one level, this could be interpreted as surrender to pro-market forces, as arguably reflected also in the ILO’s approach to labour standards following the 1998 Declaration on Fundamental Principles and Rights at Work, where various ‘core’ labour rights such as the elimination of forced labour were promoted through moral suasion rather than legal force.^122^ However, this engagement with the Bretton Woods Institutions had an upside: it allowed the ILO to promote UHC as a goal worth pursuing among those agencies of global governance historically opposed to the concept, and gently defused the political tensions that had earlier thwarted progress. This permitted UHC to return to the global political agenda, providing a fresh opportunity for concerted and coordinated international action.

Hence, despite significant challenges to its authority in the latter decades of the twentieth century, the ILO was able to press for the continued importance of SHI in an increasingly fragmented and pluralistic global health landscape, one which was often hostile to its historical aims. This repositioning came at the expense of the idealistic vision of UHC that had dominated its approach to health protection in the immediate post-war decades, an approach that revolved around the promotion of specific pathways. While the ILO was forced to adapt, and find its place in the neoliberal international order, it bought its place at the global table. This repositioning has now potentially borne fruit, both in terms of the successful achievement of UHC in many countries, and the renewed global momentum.

## 7. Conclusion

Throughout this entire period, just how successful have the ILO’s efforts to promote health protection been? At face value, the low level of ratification of social security conventions by developing countries would invite a pessimistic reply. One analysis of the ILO’s effect on national welfare spending has revealed that the ratification of conventions by developing countries is not linked in the short term to increased welfare expenditure, and ‘may be best understood as a symbolic commitment to progressive labor relations in settings where such policies face severe fiscal and organisational constraints.’^123^ As others have noted, however, the ratification of conventions is a poor gauge of the ILO’s true influence, since standard-setting is only a part of its work; what is more important is technical assistance.^124^ In this respect, this article has shown that the ILO has made a meaningful contribution to the development of health protection in countries worldwide, even if its wider role in international development has come into question in recent years.^125^ Offering advice and practical assistance to governments, and helping to design, plan, evaluate and roll-out systems in countries such as Poland and Thailand, the ILO has played an important role, one that usually goes unrecognised. This conclusion is in one sense unsurprising: As early as 1964, the political theorist Ernst Haas noted how technical assistance was often requested by low-income countries as an alternative to ratification, owing to the administrative and financial complexity of programmes. While not always ratified, ILO standards provided a normative direction of travel and inspiration for countries to follow, and influenced the course of labour legislation in regions such as Latin America.^126^ The ILO’s role in the development of health systems must not be overplayed, especially in comparison to agencies with direct responsibilities such as the WHO. However, as I have explained, the WHO has never been able to directly assist governments with logistical matters of healthcare organisation and financing, issues which are essential to sustainable health systems development. Other international agencies are also not as well placed as the ILO to dispense advice. While the ILO’s contribution to major social reforms is not always apparent, given that it largely occurs in the background and the Organisation is rarely given political credit, its agency is still visible in the patient research and technical assistance work it conducts, as well as in its continued ability to inform international thinking.

As global health leaders gather in Astana, Kazakhstan to mark the fortieth anniversary of the Alma-Ata Declaration, a new opportunity has arisen to place UHC at the front and centre of global health. The ILO also turns 100 years old in 2019: a remarkable achievement, testament to the Organisation’s ability to adapt to the changing global political landscape. It remains to be seen whether these anniversaries will provide fresh incentive for the ILO to revitalise its contribution to health protection worldwide, or whether, as in the past, the latest international momentum around UHC will fade away.

## Notes

1 Judith Rodin and David de Ferranti, ‘Universal Health Coverage: The Third Global Health Transition?’ *The Lancet*, ccclxxx, no. 9845 (2012), 861.

2 WHO, *The World Health Report 2010. Health Systems Financing: The Path to Universal Coverage* (Geneva: WHO, 2010).

3 UN, General Assembly resolution 67/81, Global Health and Foreign Policy, A/RES/67/81, 12 December 2012, available at http://undocs.org.

4 ILO, Social Floors Recommendation, 2012 (No. 202), 14 June 2012, available at http://www.ilo.org/normlex.

5 Declaration of Alma-Ata, International Conference on Primary Health Care, Alma-Ata, USSR, 6–12 September 1978, available at http://www.who.int/publications/almaata_declaration_en.pdf.

6 Marcos Cueto, ‘The Origins of Primary Health Care and Selective Primary Health Care’, *American Journal of Public Health*, xciv, no. 11 (2004), 1864–74; Randall M. Packard, *A History of Global Health: Interventions into the Lives of Other Peoples* (Baltimore: Johns Hopkins University Press, 2016).

7 WHO, *World Health Report 2010*, vi [432].

8 The Lancet, ‘The Struggle for Universal Health Coverage’, *The Lancet*, ccclxxx, no. 9845 (2012), 859.

9 Sarah Boseley, ‘From User Fees to Universal Healthcare — a 30-Year Journey’, The Guardian, 1 October 2012.

10 David de Ferranti, *Paying for Health Services in Developing Countries: An Overview*, World Bank Staff Working Papers No. 721 (Washington, DC: The World Bank, 1985).

11 Martin Gorsky and Christopher Sirrs, ‘The Rise and Fall of “Universal Health Coverage” as a Goal of International Health Politics, 1925–1952’, *American Journal of Public Health*, cviii, no. 3 (2018), 334–42.

12 ILO, Declaration Concerning the Aims and Purposes of the International Labour Organisation (Declaration of Philadelphia), 1944; ILO, Medical Care Recommendation, 1944 (No. 69), 12 May 1944, available at http://www.ilo.org/normlex.

13 Gorsky and Sirrs, ‘Rise and Fall’.

14 Daniel Maul, *Human Rights, Development and Decolonization: The International Labour Organization, 1940-70* (New York: Palgrave Macmillan, 2012).

15 Guy Standing, ‘The ILO: An Agency for Globalization?’ *Development and Change,* xxxix, no. 3 (2008), 355–84.

16 Michael Cichon, ‘The Role of the International Labour Organisation (ILO) in Health Care’, January 1992 [Geneva, Switzerland, International Labour Organisation Archive (ILOA)], SI-88 (2), 10.

17 Antony Alcock, *History of the International Labour Organisation* (London and Basingstoke: Macmillan, 1971).

18 James A. Gillespie, ‘Social Medicine, Social Security and International Health, 1940–60’, in Esteban Rodríguez-Ocaña (ed), *The Politics of the Healthy Life: An International Perspective*, History of Medicine, Health and Disease Series (Sheffield: European Association for the History of Medicine and Health Publications, 2002), 219–39.

19 Gillespie, ‘Social Medicine’; Kelley Lee, *The World Health Organization (WHO)*, Routledge Global Institutions (Abingdon: Routledge, 2008).

20 Cichon, ‘Role of the ILO in Health Care’.

21 Jill Jensen, ‘US New Deal Social Security Experts and the ILO’, 1948–1954’, in Sandrine Kott and Joëlle Droux (eds), *Globalizing Social Rights: The International Labour Organization and Beyond*, ILO Century Series (Basingstoke: Palgrave Macmillan, 2013), 172–89; Sandrine Kott, ‘Une “Communauté Épistémique” Du Social? Experts de l’OIT et Internationalisation Des Politiques Socialises Dans l’entre-Deux-Guerres’, *Genèses*, lxxi, no. 2 (2008), 26–46; Sandrine Kott, ‘Constructing a European Social Model: The Fight for Social Insurance in the Interwar Period’, in Jasmien Van Daele, Magaly Rodríguez García, Geert van Goethem, and Marcel van del Linden (eds), *ILO Histories: Essays on the International Labour Organization and Its Impact on the World during the Twentieth Century* (Bern: Peter Lang, 2010), 173–95.

22 Standing, ‘The ILO’.

23 Steve Hughes and Nigel Haworth, *The International Labour Organisation (ILO): Coming in From the Cold*, Routledge Global Institutions (Abingdon: Routledge, 2011).

24 Jensen, ‘US New Deal Social Security Experts and the ILO’; Kott, ‘Une “Communauté Épistémique” Du Social?’; Kott, ‘Constructing a European Social Model’; Gillespie, ‘Social Medicine’; Gorsky and Sirrs, ‘Rise and Fall’.

25 For the evolution of social insurance, see James A. Gillespie, ‘International Constructions of Social Security and Public Health: Policy Intersections 1920–1970’, *Gesnerus*, lxxiv, no. 2 (2017), 205–15. For variants of medical care under social security see Milton I. Roemer, *The Organisation of Medical Care under Social Security: A Study Based on the Experience of Eight Countries* (Geneva: ILO, 1969).

26 Charles Normand and Reinhard Busse, ‘Social health insurance financing’, in Elias Mossialos et al (eds), *Funding health care: Options for Europe*. European Observatory on Health Care Systems Series (Buckingham and Philadelphia: Open University Press, 2002), 59–79, 60.

27 Ibid.

28 ILO, Medical Care Recommendation, 1944; Gorsky and Sirrs, ‘Rise and Fall’.

29 Kott, 'Constructing a European Social Model'.

30 Cichon, ‘Role of the ILO in Health Care’.

31 Gorsky and Sirrs, ‘Rise and Fall’.

32 Ibid.; Jasmien Van Daele, ‘Social Protection’, in Gerry Rodgers et al (eds), *The International Labour Organization and the Quest for Social Justice, 1919–2009* (Geneva: ILO, 2009), 139–170.

33 Matthieu Leimgruber, ‘The Embattled Standard-Bearer of Social Insurance and Its Challenger: The ILO, The OECD and the “Crisis of the Welfare State”, 1975–1985’, in Sandrine Kott and Joëlle Droux (eds), *Globalizing Social Rights*: *The International Labour Organization and Beyond*, ILO Century Series (Basingstoke: Palgrave Macmillan, 2013), 293–309.

34 Maul, *Human Rights*, 140.

35 Ibid., 150–1.

36 Frederick Cooper, *Decolonization and African Society: The Labor Question in French and British Africa* (Cambridge: Cambridge University Press, 1996); Maul, *Human Rights*.

37 ILO, ‘Demande du Gouvernement de Mali’, 1966, ILOA OTA 128-4.

38 Guy Perrin, ‘Rapport au Gouvernement de La Republique Gabonaise sur la Securité Social’, 1962, ILOA OTA 261-4-1 (2).

39 Ilcheong Yi, Elizabeth Koechlein, and Armando de Negri Filho, ‘Introduction: The Universalization of Health Care in Emerging Economies’, in Ilcheong Yi (ed), *Towards Universal Health Care in Emerging Economies: Opportunities and Challenges* (London: Springer, 2017).

40 ILO, ‘Gradual Extension of Social Insurance Schemes in Latin American Countries’, International Labour Review, lxxviii, no. 3 (1958), 257, 279–80.

41 ILO, Pan American Health Organization and Permanent Inter-American Committee on Social Security, *Primary Health Care and Health Strategies in Latin American Social Security* (Geneva: ILO, 1986).

42 Albrecht Otting, ‘International Labour Standards: A Framework for Social Security’, International Labour Review, cxxxii (1993), 163–72.

43 ILO, Medical Care Recommendation, 1969 (No. 134), 25 June 1969, available at http://www.ilo.org/normlex.

44 ILO, International Labour Conference. Fifty-third session. Revision of conventions nos. 24 and 25 concerning sickness insurance. Report V (1). Geneva: ILO, 1968, 4.

45 Gillespie, ‘Social Medicine’; Gorsky and Sirrs, ‘Rise and Fall’.

46 http://www.ilo.org/normlex, accessed 16 January 2019.

47 Iris Borowy, *Coming to Terms with World Health: The League of Nations Health Organisation 1921-1946* (Frankfurt am Main: Peter Lang, 2009).

48 Gillespie, ‘Social Medicine’.

49 Ibid.

50 For evidence of ILO-WHO cooperation in the field of social security during this period, see ILOA WHO 3–3 (1). For details of joint research in relation to the rising costs of health care, see ILOA WHO 47 (1).

51 Gillespie, ‘Social Medicine’.

52 Sally Sheard, *The Passionate Economist: How Brian Abel-Smith Shaped Global Health and Social Welfare* (London: Policy Press, 2014).

53 Brian Abel-Smith, *Paying for Health Services: A Study of the Costs and Sources of Finance in Six Countries* (Geneva: WHO, 1963); ILO, *The Cost of Medical Care* (Geneva: ILO, 1959).

54 WHO, *Role of Hospitals in Programmes of Community Health Protection*. WHO Technical Report Series, No. 122 (Geneva: WHO, 1957).

55 Roemer, *Organisation of Medical Care*, 3.

56 Ibid., 23.

57 Standing, ‘The ILO’, 359.

58 Leimgruber, ‘The Embattled Standard-Bearer’.

59 WHO, ‘Minutes of informal discussion on subprogramme,’ September 1974, ILOA WHO 3-3 (2).

60 ILO and WHO, *Personal Health Care and Social Security. Report of a Joint ILO/WHO Committee* (Geneva: WHO, 1971), 30.

61 Ibid., 15, 22.

62 Milton I. Roemer and Nobuo Maeda, ‘Does Social Security Support for Medical Care Weaken Public Health Programs?’ *International Journal of Health Services*, vi, no. 1 (1976), 69–78.

63 Giovanni Tamburi to Milton Roemer, University of California Los Angeles, 14 November 1974, ILOA SI 4-01-2 (3).

64 Giovanni Tamburi, minutes, 8 December 1970, ILOA WHO 6-3-101 (2).

65 Nitsan Chorev, *The World Health Organization: Between North and South* (Ithaca: Cornell University Press, 2012); V. Djukanovic and E. P. Mach (eds), *Alternative Approaches to Meeting Basic Health Needs in Developing Countries* (Geneva: WHO, 1975).

66 WHO and UNICEF, *Primary Health Care. A Joint Report by The Director-General of the World Health Organization and The Executive Director of the United Nations Childrens’ Fund* (Geneva; New York: WHO, 1978), 42–43; WHO and UNICEF, *Primary Health Care. Report of the International Conference on Primary Health Care, Alma-Ata, USSR, 6-12 September 1978* (Geneva: WHO, 1978), 30.

67 Derick Fulcher, ‘The Primary Health Care Approach’, 1980, EASST/1980/6. Available at https://labordoc.ilo.org.

68 Richard Szal, ‘Health, Employment and Development’, 1978, ILOA WHO 1118-1 (1), 1.

69 Paul Streeten, *First Things First: Meeting Basic Human Needs in the Developing Countries* (Oxford: Oxford University Press, 1981); Gunnar Myrdal, *Asian Drama: An Inquiry into the Poverty of Nations* (Pantheon, 1968).

70 ILO, *Employment, Growth and Basic Needs: A One-World Problem* (New York: Praeger Publishers, 1977); Eddy Lee, ‘Employment and Poverty Reduction’, in Gerry Rodgers, Eddy Lee, Lee Swepston, and Jasmien Van Daele (eds), *The International Labour Organization and the Quest for Social Justice, 1919–2009* (Geneva: ILO, 2009), 171–204.

71 Streeten, *First Things First*.

72 Jacques Lemoine, note, n.d. (prob. 1978), ILOA WHO-1118-1 (1).

73 Giovanni Tamburi, ‘International Conference on Primary Health Care: USSR—September 1978’, 18 July 1978, ILOA WHO 1118-1(1), 19.

74 Fulcher, ‘Primary Health Care’, 8–9.

75 WHO, *Review of Health Expenditures, Financial Needs of the Strategy for Health for All by the Year 2000, and the International Flow of Resources for the Strategy. Report by the Director-General* (Geneva: WHO, 1981).

76 Cueto, ‘Origins’.

77 Aviva Ron, Brian Abel-Smith and Giovanni Tamburi, *Health Insurance in Developing Countries: The Social Security Approach* (Geneva: ILO, 1990), 16.

78 Surendra Jain to Halfdan Mahler (WHO Director-General), 11 January 1985, ILOA WHO 64 (1).

79 ILO, PAHO and Permanent Inter-American Committee on Social Security, *Primary Health Care and Health Strategies*.

80 PAHO, Resolution CD28.R34, Co-ordination of Social Security and Public Health Institutions, 1981, available at http://iris.paho.org.

81 ILO, PAHO and Permanent Inter-American Committee on Social Security, *Primary Health Care and Health Strategies*, 255.

82 Ibid.

83 Leimgruber, ‘The Embattled Standard-Bearer’; Peter Carroll and Aynsley Kellow, *The OECD: A Study of Organisational Adaptation* (Cheltenham: Edward Elgar, 2011); Alexandra Kaasch, *Shaping Global Health Policy: Global Social Policy Actors and Ideas about Health Care Systems* (Basingstoke: Palgrave Macmillan, 2015).

84 Leimgruber, ‘Embattled Standard-Bearer’; ILO, *Into the Twenty-First Century: The Development of Social Security* (Geneva: ILO, 1984)

85 OECD, *Health Care Systems in Transition. The Search for Efficiency*, OECD Social Policy Studies No. 7 (Paris: OECD, 1990); George J. Schieber, *Financing and Delivering Health Care: A Comparative Analysis of OECD Countries*, Social Policy Studies, No. 4 (Paris: OECD, 1987).

86 De Ferranti, *Paying for Health Services*, iv.

87 Cichon, ‘Role of the ILO in Health Care’, 14.

88 ILO, *Into the Twenty-First Century*, 65.

89 Cichon, ‘Role of the ILO in Health Care’.

90 Ibid., 15.

91 Ibid., 16; Michael Cichon and Colin Gillion, ‘Financing of Health Care in Developing Countries’, *International Labour Review*, cxxxii (1993), 174.

92 ILO, *Health Care under Social Security in Africa: Taking Stock of Experience and Potential* (Geneva: ILO, 1993), v–vi.

93 Ibid.

94 Cichon, ‘Role of the ILO in Health Care’, 16.

95 Ibid., 17.

96 Ibid.

97 Ibid., 16.

98 Michael Cichon, ‘Mission Report to First European Conference on Health Economics, Barcelona, 19-21 September 1989’, 1989, ILOA WHO 3-3 (4).

99 Michael Cichon, ‘Health Financing Strategies in Developing Countries: A Discussion Minute’, 20 November 1990, ILOA SI 88 (1).

100 WHO, ‘Study Group on the Evaluation of Recent Changes in the Financing of Health Services. Information Document’, 1991, ILOA WHO 3-3 (4).

101 Michael Cichon, ‘Note on Informal Meeting of International Agencies Involved in Health Economics’, 31 October 1991, ILOA SI-88 (1).

102 Michael Cichon, ‘Annex IV. The role of social security in health care financing in developing countries. Notes for presentation at the World Bank, 22 September 1992’, ILOA SI 88 (2), 8–9.

103 A. Seth-Mani, ‘Aide-memoire of ILO-WHO Meeting, 25 September 1984’, 1984, ILOA WHO 64 (1).

104 Colin Gillion, ‘Re: Draft: Strategies to Improve the Economic and Financial Basis of Health Care Systems: Joint Statement by the ILO and WHO Executive Heads’, 10 September 1991, ILOA SI 88 (1); ILO and WHO, ‘Joint Statement on Strategies to Improve the Economic and Financial Basis of Health Care Systems (Draft)’, 16 May 1991, ILOA SI 88 (1), 1, 3.

105 Michael Cichon, ‘Re: Future Collaboration with WHO’, 11 January 1991, ILOA SI 88 (1); Oral history interview, November 2017.

106 Cichon, ‘Re: Future Collaboration with WHO’.

107 Charles Normand and Axel Weber, *Social Health Insurance: A Guidebook for Planning* (Geneva: WHO), 1994.

108 Michael Cichon, ‘Health Sector Reforms in Central and Eastern Europe: Paradigm Reversed?’, *International Labour Review*, cxxx, no. 3 (1991), 311–27.

109 Michael Cichon, ‘Notes, Observations and Comments on Health Insurance Financing of Health Services in Poland’, 4 December 1992, ILOA SI 2-0-50, 14.

110 Ibid.

111 ILO, ‘ILO Contribution to the Polish Social Strategy Study’, 29 September 1992; ILOA SI 2-0-50 (4).

112 Gillion, ‘Re: Draft: Strategies’.

113 Cichon, ‘Role of the ILO in Health Care’.

114 Ibid.; ILO and UNDP, *Federal Republic of Nigeria. Implementation of a National Health Insurance Scheme: Phase 1* (Geneva: ILO, 1993).

115 ILO, ‘Agency Report for the “Inter-Agency Consultations on Health Economics and Financing"’, 31 October 1991, ILOA SI 88 (1); ILO, *Thailand. Review of the Social Security Scheme. Part 1: Summary and recommendations* (Geneva: ILO, 1998).

116 Erik Martinez Kuhonta, ‘The Politics of Health Care Reform in Thailand’, in Ilcheong Yi (ed), *Towards Universal Health Care in Emerging Economies: Opportunities and Challenges* (London: Springer, 2017), 91–118, 93.

117 Thaworn Sakunphanit, *Universal Health Care Coverage through Pluralistic Approaches: Evidence from Thailand* (Bangkok: ILO Subregional Office for East Asia, 2008).

118 Monica Burns, ILO consultant, oral history interview, September 2017.

119 Joseph Harris, ‘“Developmental Capture” Of the State: Explaining Thailand’s Universal Coverage Policy’, *Journal of Health Politics, Policy and Law* xl, no. 1 (1 February 2015): 165–93, 181.

120 Ibid.; Cichon, interview, 2017.

121 ILO, *Social Health Protection: An ILO Strategy towards Universal Access to Health Care*, Social Security Policy Briefings. Paper 1 (Geneva: ILO, 2008).

122 Hughes and Haworth, *The International Labour Organisation (ILO)*; Standing, ‘The ILO’, 355–84; Anne-Emanuelle Birn, Laura Nervi, and Eduardo Siqueira, ‘Neoliberalism Redux: The Global Health Policy Agenda and the Politics of Cooptation in Latin America and Beyond’, *Development and Change*, xlvii, no. 4 (2016): 734–59.

123 David Strang and Patricia Mei Yin Chang, ‘The International Labor Organization and the Welfare State: Institutional Effects on National Welfare Spending, 1960-80’, *International Organization*, xlvii, no. 2 (1993), 250.

124 Sandrine Kott and Joëlle Droux, eds. ‘Introduction: A Global History Written from the ILO’, *Globalizing Social Rights: The International Labour Organization and Beyond*, ILO Century Series (Basingstoke: Palgrave Macmillan, 2013), 1–16.

125 Standing, ‘The ILO’.

126 Ernst B. Haas, *Beyond the Nation-State: Functionalism and International Organization* (Stanford: Stanford University Press, 1964).

